# Risk perception regarding the COVID-19 outbreak among the general population: a comparative Middle East survey

**DOI:** 10.1186/s43045-020-00080-7

**Published:** 2020-12-22

**Authors:** Mahmoud Abdel Hameed Shahin, Rasha Mohammed Hussien

**Affiliations:** 1 Al-Ghad International Colleges for Applied Medical Sciences, Qassim, Saudi Arabia; 2 Department of Psychiatric and Mental Health Nursing, Faculty of Nursing, Zagazig University, Zagazig, Egypt

**Keywords:** Coronavirus, Efficacy, Precautions, Preventive measures, Seriousness perception

## Abstract

**Background:**

People’s perceptions of pandemic-associated risk are key factors contributing to increased public participation in disease preventive measures. The aim of the study was to investigate risk perceptions regarding the coronavirus disease 2019 (COVID-19) outbreak, among the general population. A descriptive, cross-sectional design was used with a convenience sample of 723 participants, recruited from the general population of Saudi Arabia, Egypt, and Jordan. Data collection was performed using a standardized risk perception assessment questionnaire, in April 2020.

**Results:**

The mean score for the perception of COVID-19 seriousness was significantly higher and the mean scores for the perception of disease susceptibility and extent of anxiety were also higher among Saudi Arabian participants than participants from Egypt and Jordan. Participants from Egypt had significantly lower mean scores for the perception of efficacy and self-efficacy to cope with COVID-19, and significantly lower intention to comply with COVID-19 precautionary measures than the other populations. A significant positive correlation was detected between the perception of COVID-19 seriousness and self-efficacy to handle COVID-19, for the entire sample. The primary reasons reported by participants driving their willingness to perform certain preventive measures against COVID-19 was a feeling of responsibility toward their own health, followed by preventing transmission to other people and the feeling that COVID-19 can be serious. Most of the study sample reported a desire to receive information about COVID-19 treatment, ways to prevent disease contraction, and the incubation period for the novel coronavirus. Also, most of the study sample reported that they prefer receiving COVID-19 updates from national authorities.

**Conclusions:**

During the COVID-19 pandemic, communications designed to promote the adoption of preventive behaviors should focus on increasing the perception of seriousness, the risk perception, self-efficacy to cope with the COVID-19 pandemic, and the effectiveness of the adopted behavioral measures for reducing risk. Health education programs that are tailored to various sociodemographic categories, to improve public awareness, perceptions, and attitudes, are vital for increasing the adoption of outbreak preventive measures.

## Background

The coronavirus disease 2019 (COVID-19) outbreak, caused by severe acute respiratory syndrome coronavirus 2 (SARS-CoV-2), appears to have originated in Wuhan, China, in December 2019 [1]. Subsequently, it has spread dramatically, both inside and outside of China [2], and has grown to become an exceptional, global, public health problem [3].

In the context of the novel coronavirus outbreak in 2020, the world is racing to contain the spread of the lethal virus, worldwide. At present, the fatality rate associated with COVID-19 is lower than those for previous epidemics, such as SARS and Middle East respiratory syndrome (MERS). COVID-19 transmission occurs rapidly, through direct human-to-human contacts, which has made effectively informing the public regarding the risks and necessary precautions for transmission prevention difficult to achieve [4]. On 30 January 2020, the World Health Organization (WHO) declared the outbreak of COVID-19 to be a global public health emergency. Following the exponential growth observed in the numbers of infected cases and affected countries, COVID-19 was consequently declared to be a pandemic on 11 March 2020 [5, 6].

COVID-19 appears to present unique epidemiological features compared with the previous SARS-CoV outbreak. COVID-19 replicates efficiently in the human upper respiratory tract of humans, results in the less abrupt onset of symptoms, and mimics the conventional human coronaviruses, which are the predominant causes of common colds during winter [7]. Infected humans produce and replicate massive amounts of the virus in the upper respiratory tract during a prodromal period, during which time the infected individuals are generally actively mobile and can continue to perform their usual activities, which has contributed to rapid and widespread infections. In contrast, the transmission of SARS-CoV was not verified to be able to occur during the prodromal period, when infected patients report only mild illness, and most transmissions were confirmed to occur when the infected individuals presented with symptoms and were extremely ill, likely resulting in SARS-CoV outbreaks being easier to contain than modern-day outbreaks associated with COVID-19 [8].

According to the Saudi Center for Disease Prevention and Control (SCDC), the total number of confirmed cases in Saudi Arabia has reached 157,612, and the total number of deaths has reached 1267, as of 22 Jun 2020 [9]; in Egypt, the total number of confirmed cases was 55,233, and the total number of deaths was 2193; and in Jordan, 1033 confirmed cases and 9 total deaths were reported, for the same time point [10].

In the absence of available pharmaceutical protocols to treat affected persons or existing vaccines to control infections, most countries have implemented containment and mitigation strategies, requiring people to dramatically alter their life styles and limiting their personal freedom. The extent to which these measures are practical, suitable, and applicable for the population depends upon other factors, including individuals’ perceptions of the threat of suffering negative and dangerous health consequences associated with the infection [11]. Not all persons are at equal risk of death if infected. However, present records from affected areas have shown that individuals with a pre-existing medical history of chronic illnesses and older people are at higher risk of experiencing death as a consequence of COVID-19 [12].

Following the WHO declaration, many countries, including the Kingdom of Saudi Arabia, have implemented response plans to counter the pandemic and contain the viral spread. Following the confirmation of its first COVID-19 infection, on Monday, 2 March 2020, Saudi Arabian authorities have been vigilantly monitoring the situation and deciding which country-specific measures to employ to comply with WHO instructions for handling an outbreak [13]. These measures have encompassed the suspension of all inbound and outbound flights, closing all shops, malls, and stores in the country except for pharmacies and grocery stores, and closing down all educational institutions, schools, and universities. Umrah visas for Muslims have been suspended, as have prayers at mosques including the two Holy Mosques in Makkah and Al-Madina. On 24 March 2020, the Saudi Arabian authorities imposed a nationwide curfew to limit gathering and movement during daytime hours [13].

To date, the global scenario appears to be under control. However, the socioeconomic circumstances in Africa are extremely less well-developed and often worse than in other nations. A collection of factors, including the shortage of medical supplies, a lack of medical services, poor general health conditions, and decreased virus testing efficiency, may facilitate the spread of the epidemic in African countries. Additionally, the dry season in Africa is approaching, and the abrupt drop in daily average temperatures may be conducive to enhancing the spread of the disease. Consequently, a severe COVID-19 outbreak in Africa may occur if no strict preventive measures and management plans are implemented in these countries [14].

To combat the spread of COVID-19, various preventive and control measures, at different levels, have been employed by various countries, around the world. Among these measures, on the individual level, the Jordanian Ministry of Health has stipulated the maintenance of at least 6 ft of social distance between individuals, frequent hand-washing, the avoidance of face touching after touching surfaces, the frequent use of hand sanitizers and alcohol hand rubs, practicing coughing and sneezing etiquette, avoiding handshaking and kissing, avoiding direct contacts with sick persons, particularly these who demonstrate the signs and symptoms of respiratory infections, and wearing face masks under specific conditions [15].

Risk perception is a central characteristic of many health-behavior theories. According to the Protection Motivation Theory, protection motivation is a consequence of risk or threat assessment and coping appraisal. Threat assessment consists of estimating the hazard of contracting a disease (perceived vulnerability or susceptibility) and estimating the seriousness of a disorder or sickness (perceived severity) [16]. The personal perception of infection threat is a key issue that influences the spread of epidemics, to obtain realistic inferences, epidemiological models must consider such parameters [17]. However, increased hazard or risk perceptions may only predict defensive behaviors, particularly when human beings believe that effective responses and defensive actions are readily available (response efficacy) and when they are confident in their competencies to interact and engage in such defensive actions (self-efficacy) [18, 19].

According to Slovic and Peters [20], two exclusive types of risk perceptions exist, affective and cognitive responses, which may exhibit extraordinary patterns of alterations and changes over the course of public health emergencies. Affective responses have been described as the emotional responses to risk, and cognitive elements encompass the perceived seriousness of the threat and an individual’s perceived coping efficacy [20]. Cameron and Leventhal [21] suggested that the affective response is experiential, rapid, and intuitive, whereas the cognitive response is deliberate, slow, and rule-based [21].

Furthermore, during the early stages of a potential pandemic, such as the COVID-19 outbreak, risk perceptions and efficacy beliefs are established in response to communications with and between individuals in the groups at risk. Risk communication messaging that is not perceived or comprehended by the public or the communication of conflicting threat messaging is likely to result in the limited embrace of precautionary interventions by the public. Communications that are perceived as coming from a non-trustworthy resource can also have similar consequences. However, risk communication messaging can be very quickly adopted by the media, resulting in the “amplification” of threat and hazard information, which can also lead to unnecessary scares and the adoption of useless or ineffective precautionary actions or responses by the public [22].

In an effort to combine over 50 years of risk perception research, the Van der Linden risk perception model recommended the inclusion of clusters of variables that are closely related to the cognitive tradition (e.g., comprehension and perceptions of risks), the emotional and experiential tradition (e.g., private, personal experiences), the social-cultural paradigm (e.g., the social amplification of risks, cultural theories, trust, and values), and relevant individual variations (e.g., gender, education, and ideology) [23, 24]. This “holistic” approach to modeling the determinants of risk perception can prevent the overreliance on any single paradigm, alleviating concerns regarding the questionable reliability of single-item constructs, and has been adopted in recent studies of disease outbreaks [25]. Therefore, some studies have measured risk perception using an index that encompasses the cognitive (likelihood), emotional (concern and worry), and temporal-spatial dimensions of risk [26, 27].

### Significance of study

The health emergencies associated with the COVID-19 pandemic have had major impacts on the safety and health of societies, at the local and international levels. Community cooperation in the implementation of health preventive measures, through adherence to instructions and recommendations issued by the WHO and other government agencies, represents a cornerstone for reducing the spread of the virus and reducing the long-term impacts of this pandemic on public health. As the numbers of confirmed cases and deaths associated with the COVID-19 pandemic continues to increase, around the world, understanding public risk perceptions has become very important. Threat appraisal and risk perception are known to be important determinants of the public’s willingness to cooperate with and adopt health-protective behaviors during pandemics.

Risk perception plays a major role in estimating the extent of community awareness regarding the seriousness of this pandemic and the extent of the willingness to cooperate in the implementation of health preventive measures, at the individual, local, and international levels. A research gap exists regarding the extent of risk perception in response to health pandemics among various societies, especially Arab societies. Additionally, the identification of the level of awareness among society members regarding the COVID-19 pandemic, their intentions and abilities to apply preventive measures, and their sense of self-efficacy in the application of recommended measures represent baseline levels of information that must be gathered during scientific research to determine how best to optimize community awareness through scientific interventions, media releases, and government channels. By collecting this information, we can determine how to increase the effectiveness of societal interventions designed to minimize the effects of the COVID-19 pandemic and identify methods for improving societal cooperation and reactions to future pandemic situations.

Therefore, assessing risk perception and self-efficacy during the course of the current COVID-19 outbreak has been deemed imperative. We aimed to collect a large sample of the population and utilized a comparative study design to examine countries in the Middle East region that are accessible to the researchers including Egypt, Jordan, and Saudi Arabia. Saudi Arabia is a large country located in Western Asia and forming the bulk of the Arabian Peninsula, with a population of 34 million, as reported in 2019 [28]. Jordan is a small Arab country also located in Western Asia on the East Bank of the Jordan River, with a population of more than 10 million, as reported in 2020 [29]. Egypt is an African Arab country, with over 100 million inhabitants, as reported in 2020 [30]. All three countries are considered to be located in the Middle East region.

### Aim of the study

This study aimed to investigate the risk perceptions regarding the COVID-19 outbreak among the general populations of Saudi Arabia, Jordan, and Egypt.

### Research question

Are there differences in risk perceptions among the general populations of Saudi Arabia, Egypt, and Jordan?

## Methods

### Research design and settings

A descriptive, cross-sectional, comparative research design was used in this study. This study was performed among a sample drawn from the general populations of Saudi Arabia, Egypt, and Jordan. These three countries were chosen because of their geographical proximity, easy accessibility, and the presence of researchers in these countries facilitated the data collection process.

### Participants

The sample size was calculated according to the proportional populations of the three countries, at a 99% confidence level, with 5% confidence limits, 50% anticipated frequency, and a design effect value of 1.0. Using the Open-Epi, version 3.01 software package, the required sample size was determined to be a minimum of 664 subjects [31]; however, it was increased to ensure the achievement of the targeted confidence level. A convenience sample of 723 individuals from among the general population was recruited for this comparative study, from Saudi Arabia (468 participants), Egypt (162 participants), and Jordan (93 participants). The criteria for inclusion were: being resident in one of the three included countries, being adult, able to read Arabic, and willing to participate in the study.

### Data collection tool

The data collection questionnaire consisted of two sections. The first section consisted of a sociodemographic data collection sheet, which included age, gender, marital status, and educational level.

The second section was the “Standard questionnaire on risk perception of an infectious disease outbreak,” which was designed by the Municipal Public Health Service Rotterdam-Rijnmond, together with the National Institute for Public Health and the Environment in the Netherlands [32]. This tool was designed to study risk perceptions associated with the outbreak of infectious disease and has been used in many previous studies [33, 34]. The generic questionnaire was tailored to address COVID-19 and was then translated into Arabic, which is the predominant language of the study sample in the chosen countries. The questionnaire opened with an introduction, which informed the respondents of the study objective and provided instructions for questionnaire completion and submission. The questionnaire covered the following seven domains:

1. Participant’s knowledge, which was assessed by one question regarding the availability of a COVID-19 vaccine and was answered as yes, no, or I don’t know.
2. Perception of the seriousness of COVID-19, which consisted of two questions, with responses based on a 5-point Likert scale (ranging from 1 = not at all serious to 5 = very serious).
3. Perception of susceptibility to COVID-19 and the extent of anxiety, which was assessed by three questions, using a 5-point Likert scale ranging from 1 to 5, with higher scores indicating greater anxiety regarding COVID-19.
4. Perception of efficacy and self-efficacy in dealing with COVID-19, which consisted of eight items, assessed by a 5-point Likert scale, ranging from 1 = certainly not to 5 = most certainly.
5. Intention to perform preventive measures against COVID-19, assessed using one item, which was scored on a 5-point Likert scale, ranging from 1 = certainly not to 5 = most certainly.
6. Motivating/hindering factors affecting the intention to perform preventive measures, which were assessed using two questions that probed the reasons underlying a participant’s willingness and unwillingness to employ preventive measures. Both questions included many choices, and the participants were allowed to select up to three answers for each question.
7. Information needs assessment, which consisted of three questions asking which important topics the participants desire additional information on, where they would prefer to obtain this information, and how they would prefer to receive this information. These questions featured many choices, and the participants were allowed to select up to three answers for each question.

### Pilot study

A pilot study was performed before executing the main study, to test the clarity of the scales and the feasibility of the study. Utilizing the same inclusion criteria of the study, the pilot study was conducted on 10% of the estimated study sample (66 subjects), to test the applicability of the data collection tool and the feasibility of the study. Based on the pilot study results, the average time required to respond to the questionnaire ranged from 15 to 20 min, depending on the respondent’s level of understanding and cooperation. Based on the pilot study results, the questionnaire was finalized. Because some modifications were made to the phrasing of some of the questionnaire items, the pilot study subjects were not included in the main study sample. Participants included in piloting were informed that they will not be allowed to participate in the study if their responses are excluded later. Furthermore, the pilot study was used to assess the reliability of the questionnaire.

### Validity and reliability

The questionnaire was reviewed by a panel of five experts in the fields of psychiatric mental health nursing, public health, and medical-surgical nursing, to test the content and face validity of the questionnaire, which were deemed acceptable. Reliability was determined using a test-retest comparison, and the calculation of Cronbach’s alpha revealed acceptable reliability (Cronbach’s alpha = 0.814).

### Procedure

Similar to other countries, around the world, the Saudi Arabian, Egyptian, and Jordanian governments have recommended that the public minimize face-to-face interactions and isolate themselves at home; therefore, potential respondents were invited to participate in the study remotely, using a Google Forms-prepared questionnaire. The survey was sent to interested participants via various applications (WhatsApp, Messenger, and Imo), and generally completed the online survey in 15 to 20 min. The study and all data handling processes followed all national required data protection standards. Participants were given the option of providing an email address to receive a summary of the study findings, and all participants provided informed consent before starting the questionnaire. The study did not include deception, and participants were debriefed at the end of the survey. Data collection occurred over 15 days (16 April 2020 to 30 April 2020), after the WHO declared the COVID-19 outbreak to be a public health emergency of international concern. The duration of data collection was sufficient to gather the predetermined sample size.

### Statistical analysis

Data retrieved from Google Forms were collected, revised, and coded, using a personal computer (PC). Statistical analysis was performed using the Statistical Package for Social Sciences (SPSS), version 23 [35]. Data are presented using descriptive statistics, as the mean and standard deviation or number and percentage. The mean and standard deviation were used for continuous variables, whereas the number and percentage were used for categorical variables. No missing data were expected as all questions were mandatory to be answered before moving to the next question. The Shapiro-Wilk test indicated that the data were not normally distributed; therefore, the Kruskal-Wallis test was used to compare the mean scores for participants’ perceptions of “seriousness,” “susceptibility,” “self-efficacy,” and “intention to perform preventive measures” in response to coronavirus across the three countries. The Mann-Whitney *U* test was used to identify significant differences in mean scores between each pair of two countries, and to compare means according to dichotomous sociodemographic characteristics, such as gender and marital status. The Mann-Whitney *U* test and the Kruskal-Wallis test are nonparametric tests that can be used to compare mean rank values but cannot be used to compare medians or distributions. Pearson’s correlation coefficient test was used to examine the correlation between the mean scores for the perception of coronavirus “seriousness” and “self-efficacy” among participants, with statistical significance was set at *P* < .05. However, the Open-Epi software package was used to estimate the required sample size of the study.

### Ethical considerations

The research proposal was submitted to the ethical committee at Al-Ghad International Colleges for Applied Medical Sciences in Saudi Arabia and was approved prior to commencing data collection. In addition, after reading the introductory information about the study, participants were asked to provide informed consent, before starting the questionnaire. The online survey form contained text regarding informed consent, which was included in the questionnaire and approved by all participants. Anonymity and confidentiality were promoted through the use of survey identification numbers and an anonymous questionnaire, with no collection of personal identifiers, such as names, phone numbers, or other information that could be used to identify participants or link participants to data. No risk of discomfort to the participant was anticipated, other than the potential inconvenience associated with the time necessary to participate in the study, and participants did not receive any form of coercion or financial compensation, as participation was voluntary, with full autonomy. All ethical principles regarding medical research involving human subjects were followed, in accordance with the Declaration of Helsinki [36]. Additionally, official permission to use and modify the questionnaire was granted by the original authors [32].

## Results

Table 1 shows the sociodemographic characteristics of the study sample (723 participants) who were recruited from three Arab countries (Saudi Arabia, Egypt, and Jordan), which demonstrates that most of the participants were aged from 30 to 39 years (42.4%), almost two-thirds were male (63.5%), and more than three-quarters were married (76.3%). Approximately half of the participants from Saudi Arabia and Jordon held bachelor’s degrees (49.4% and 51.6%, respectively), and nearly one-third of participants from these countries also engaged in postgraduate studies, whereas 50% of Egyptian participants held technical diplomas and only slightly more than one-quarter (27.8%) held bachelor’s degrees.

**Table 1:** Sociodemographic characteristics of the study sample (*N* = 723)

Sociodemographics	Saudi Arabia (468)		Egypt (162)		Jordan (93)		Total	
	*n*	%	*n*	%	*n*	%	*n*	%
Age								
Younger than 24 years	72	15.4%	39	24.1%	3	3.2%	114	15.8%
25–29 years	51	10.9%	21	13.0%	9	9.7%	81	11.2%
30–34 years	99	21.2%	24	14.8%	9	9.7%	132	18.3%
35–39 years	120	25.6%	21	13.0%	33	35.5%	174	24.1%
40–44 years	81	17.3%	24	14.8%	15	16.1%	120	16.6%
45–49 years	27	5.8%	12	7.4%	15	16.1%	54	7.5%
50 years and older	18	3.8%	21	13.0%	9	9.7%	48	6.6%
Gender								
Female	183	39.1%	63	38.9%	18	19.4%	264	36.5%
Male	285	60.9%	99	61.1%	75	80.6%	459	63.5%
Marital status								
Single	117	25.0%	33	20.4%	21	22.6%	171	23.7%
Married	351	75.0%	129	79.6%	72	77.4%	552	76.3%
Educational level								
School	24	5.1%	24	14.8%	6	6.5%	54	7.5%
Diploma	87	18.6%	81	50.0%	12	12.9%	180	24.9%
Bachelor’s degree	231	49.4%	45	27.8%	48	51.6%	324	44.8%
Postgraduate studies	126	26.9%	12	7.4%	27	29.0%	165	22.8%

*n* frequency, % percentage

Table 2 shows that three-quarters of all participants reported that no vaccine against the COVID-19 exists, to date (75.1%).

**Table 2:** Knowledge about COVID-19 vaccine (*N* = 723)

Knowledge about COVID-19 vaccine	*n*	%
Is there a vaccine against COVID-19?		
No	543	75.1%
Yes	45	6.2%
Don't know	135	18.7%

*n* frequency, % percentage

Table 3 reveals that most of the study sample perceived the COVID-19 pandemic to be either a serious or most serious event (44.4% and 43.6%, respectively). Furthermore, 40.7% of participants perceived the possibility of contracting COVID-19 in the near future to represent a serious event, whereas 32.8% viewed this possibility as very serious.

**Table 3:** Perceptions of the seriousness of COVID-19 (*N* = 723)

Perception of the seriousness of COVID-19	*n*	%
How serious do you think COVID-19 is?		
Not at all serious	3	0.4%
Not serious	9	1.2%
Slightly serious	75	10.4%
Serious	321	44.4%
Very serious	315	43.6%
How would you feel if you contracted COVID-19 in the near future?		
Not at all serious	6	0.8%
Not serious	51	7.1%
Slightly serious	135	18.7%
Serious	294	40.7%
Very serious	237	32.8%

*n* frequency, % percentage

The Shapiro-Wilk test was performed to determine the normality of the data, and the findings revealed that not all variables were normally distributed, based on the demographic characteristics (*P* < .01). Therefore, non-parametric tests were used to detect significant differences in the mean scores. Table 4 demonstrates that participants from Saudi Arabia reported a significantly higher mean perception of seriousness than those from Egypt and Jordan (*P* < .05).

**Table 4:** Difference in mean “perception of the seriousness of COVID-19” scores, based on the country of origin

Perception of the seriousness of COVID-19	Saudi Arabia	Egypt	Jordan	Total	Kruskal-Wallis test	*P* value
	*n* = 468	*n* = 162	*n* = 93	*N* = 723		
How serious do you think COVID-19 is?						
M ± SD	4.42 ± 0.631	4.11 ± 0.788	4 ± 0.956	4.29 ± 0.735	Chi-square = 28.941742	< .001**
Mean rank	389.96	315.11	302.97			
How would you feel if you contracted COVID-19 in the near future?						
M ± SD	4.03 ± 0.917	3.96 ± 0.905	3.71 ± 1.028	3.98 ± 0.934	Chi-square = 8.221587	.016*
Mean rank	374.03	356.86	310.42			
Mean perception of seriousness scores						
M ± SD	4.224 ± 0.68	4.037 ± 0.74	3.854 ± 0.88	4.134 ± 0.73	Chi-square = 18.326	< .001**
Mean rank	384.7019	333.44	297.5			

*M* mean, *SD* standard deviation

*Significant at the .05 level (two-tailed)

**Significant at the .01 level (two-tailed)

Table 5 demonstrates that near half of the study sample reported “perhaps yes-perhaps no” for their perception of the likelihood of contracting COVID-19 in the coming year if they did not take any preventive measures (47.7%), whereas 20.3% reported that they “most certainly” would contract COVID-19. More than two-thirds of participants responded either “neither small chance, nor large” or “large chance” for their perceptions of the likelihood of contracting COVID 19 in the coming year without a vaccine (39.8%, and 31.5% respectively), and slightly more than two-thirds reported being either “slightly concerned” or “concerned” about the possibility of contracting COVID-19 (36.9% and 34.9%, respectively).

**Table 5:** Perceptions of susceptibility to COVID-19 and the extent of anxiety (*N* = 723)

Perception of susceptibility to Coronavirus COVID-19 and extent of anxiety	*n*	%
Do you think that you will contract COVID-19 in the coming year if you do not take any preventive measures?		
Certainly not	27	3.7%
Probably not	63	8.7%
Perhaps yes-perhaps no	345	47.7%
Probably yes	141	19.5%
Most certainly	147	20.3%
Suppose you have not been vaccinated against COVID-19 or the vaccine is not available. What do you think your chance of contracting the disease in the coming year is?		
Very small chance	60	8.3%
Small chance	90	12.4%
Not small - not large	288	39.8%
Large chance	228	31.5%
Very large chance	57	7.9%
How concerned are you about contracting COVID-19?		
Not at all concerned	30	4.1%
Not concerned	48	6.6%
Slightly concerned	267	36.9%
Concerned	252	34.9%
Very concerned	126	17.4%

*n* frequency, % percentage

Comparing the mean “perception of susceptibility to COVID-19” scores across countries (Table 6) showed that Saudi Arabian participants reported significantly higher mean scores regarding the likelihood of contracting COVID-19 in the absence of preventive measures compared with participants from Egypt and Jordan (*P* < .05), reflecting generally a higher perception of susceptibility and anxiety to COVID-19 for respondents from Saudi Arabia.

**Table 6:** Differences in mean “perception of susceptibility to COVID-19 and the extent of anxiety” scores, according to country (*N* = 723)

Perception of susceptibility to Coronavirus	Saudi Arabia	Egypt	Jordan	Total	Kruskal-Wallis test	*P* value
	*n* = 468	*n* = 162	*n* = 93	*N* = 723		
Do you think that you will contract COVID-19 in the coming year if you do not take any preventive measures?						
M ± SD	3.51 ± 1.125	3.29 ± 0.713	3.35 ± 0.939	3.44 ± 1.0260	Chi-square = 6.041	.049*
Mean rank	374.81	333.11	347.87			
Suppose you have not been vaccinated against Coronavirus COVID-19 or the vaccine is not available. What do you think your chance of contracting the disease in the coming year is?						
M ± SD	3.23 ± 1.075	3.21 ± 0.849	3.17 ± 1.074	3.18 ± 1.027	Chi-square = 0.030	.985
Mean rank	362.87	361.05	359.29			
How concerned are you about contracting COVID-19?						
M ± SD	3.55 ± 0.977	3.67 ± 0.946	3.32 ± 1.095	3.55 ± 0.989	Chi-square = 5.518	.063
Mean rank	361.8	384.47	323.87			
Mean perception of susceptibility to COVID-19 and the extent of anxiety						
M ± SD	3.41 ± 0.765	3.38 ± 0.603	3.30 ± 0.878	3.39 ± 0.748	Chi-square = 1.827	.401
Mean rank	368.30	358.00	337.27			

*M* mean, *SD* standard deviation

*Significant at the .05 level (two-tailed)

Table 7 demonstrates that slightly more than two-thirds of the study sample were most certain that frequent hand hygiene helps to prevent COVID-19 infections and that they will wear masks and practice social distancing, if advised (66.8%, 61.0%, and 67.6%, respectively). The majority of the study sample also responded that maintaining social distancing and implementing quarantine most certainly helps to prevent COVID-19 infection (73.0% and 78.4%, respectively). Additionally, more than two-thirds reported that they will implement hand hygiene and quarantine protocols, if advised (75.9% and 72.2%, respectively).

**Table 7:** Perceptions of efficacy and self-efficacy in dealing with COVID-19 (*N* = 723)

Perception of efficacy and self-efficacy	Certainly not		Probably not		Perhaps yes-perhaps no		Probably yes		Most certainly	
	*n*	%	*n*	%	*n*	%	*n*	%	n	%
1. Do you think that frequent hand hygiene helps to prevent COVID-19?	21	2.9%	9	1.2%	75	10.4%	135	18.7%	483	66.8%
2. Do you think that wearing masks helps to prevent COVID-19?	33	4.6%	42	5.8%	174	24.1%	243	33.6%	231	32.0%
3. Do you think that maintaining social distancing helps to prevent COVID-19?	3	0.4%	6	0.8%	48	6.6%	138	19.1%	528	73.0%
4. Do you think that quarantine helps to prevent COVID-19?	0	0.0%	9	1.2%	45	6.2%	102	14.1%	567	78.4%
5. Do you think that you will manage to implement hand hygiene, if this is advised?	3	0.4%	9	1.2%	48	6.6%	114	15.8%	549	75.9%
6. Do you think that you will manage to implement mask-wearing, if this is advised?	0	0.0%	27	3.7%	84	11.6%	171	23.7%	441	61.0%
7. Do you think that you will manage to implement social distancing, if this is advised?	3	0.4%	15	2.1%	57	7.9%	159	22.0%	489	67.6%
8. Do you think that you will manage to implement quarantine, if this is advised?	0	0.0%	6	0.8%	60	8.3%	135	18.7%	522	72.2%

*n* frequency, % percentage

Table 8 shows that the population sample from Saudi Arabia scored significantly higher in the items “Do you think that you will manage to implement mask-wearing?”, “Do you think that you will manage to implement social distancing?”, and “Do you think that you will manage to implement quarantine?” compared with the population samples from the other two countries (*P* < .01), whereas the population sample from Jordan scored significantly higher in the item “Do you think that wearing masks helps to prevent COVID-19?” compared with the population samples from the other two countries. The population sample from Egypt scored the lowest in all items of self-efficacy for dealing with COVID-19, and they also scored significantly lower in the items examining their beliefs that frequent hand hygiene, social distancing, and quarantine help to prevent COVID-19 and in the item examining their ability to maintain hand hygiene. Additionally, the Egyptian participants had significantly lower mean self-efficacy total scores than participants from Jordan and Saudi Arabia (*P* < .01).

**Table 8:** Differences in mean “perception of efficacy and self-efficacy” scores, according to country of origin

Perception of efficacy and self-efficacy	Saudi Arabia	Egypt	Jordan	Total	Kruskal-Wallis test	*P* value
	*n* = 468	*n* = 162	*n* = 93	*N* = 723		
1. Do you think that frequent hand hygiene helps to prevent COVID-19?						
M ± SD	4.56 ± 0.936	4.07 ± 0.902	4.55 ± 0.841	4.45 ± 1.938	Chi-square = 68.700	< .001**
Mean rank	393.96	262.94	373.71			
2. Do you think that wearing masks helps to prevent COVID-19?						
M ± SD	3.83 ± 1.104	3.61 ± 0.934	4.16 ± 1.145	3.83 ± 1.084	Chi-square = 25.954	< .001**
Mean rank	365.02	308.44	440.1			
3. Do you think that maintaining social distancing helps to prevent COVID-19?						
M ± SD	4.80 ± 0.549	4.09 ± 0.825	4.74 ± 0.509	4.63 ± 0.682	Chi-square = 166.683	< .001**
Mean rank	408.02	217.97	381.31			
4. Do you think that quarantine helps to prevent COVID-19?						
M±SD	4.82 ± 0.561	4.31 ± 0.768	4.74 ± 0.509	4.70 ± 0.641	Chi-square = 110.845	< .001**
Mean rank	398.96	255.17	362.10			
5. Do you think that you will manage to implement hand hygiene, if this is advised?						
M ± SD	4.80 ± 0.525	4.20 ± 0.953	4.71 ± 0.582	4.6 6± 0.695	Chi-square = 93.544	< .001**
Mean rank	396.31	258.92	368.92			
6. Do you think that you will manage to implement mask-wearing, if this is advised?						
M ± SD	4.56 ± 0.727	4.04 ± 0.984	4.35 ± 0.868	4.42 ± 0.837	Chi-square = 49.050	< .001**
Mean rank	394.11	279.14	344.77			
7. Do you think that you will manage to implement social distancing, if this is advised?						
M ± SD	4.73 ± 0.571	4.09 ± 1.026	4.39 ± 0.708	4.54 ± 0.762	Chi-square = 84.597	< .001**
Mean rank	404.69	269.50	308.29			
8. Do you think that you will manage to implement quarantine, if this is advised?						
M ± SD	4.79 ± 0.520	4.26 ± 0.846	4.42 ± 0.712	4.62 ± 0.672	Chi-square = 92.419	< .001**
Mean rank	404.86	272.89	301.56			
Total self-efficacy scores						
M ± SD	4.6130 ± 0.4762	4.0856 ± 0.6541	4.5081 ± 0.4329	4.4813 ± 0.5589	Chi-square = 99.540	< .001**
Mean rank	412.2	224.31	349.23			

*M* mean, *SD* standard deviation

**Significant at the .01 level (two-tailed)

Table 9 showed that the majority of the study sample (78.0%) answered “most certainly” in response to their intention to implement advised preventive measures against the new coronavirus.

**Table 9:** Intention to implement preventive measures against COVID-19

Intention to implement preventive measures	Saudi Arabia (*n* = 468)		Egypt (*n* = 162)		Jordan (*n* = 93)		Total (*N* = 723)	
	*n*	%	*n*	%	*n*	%	*n*	%
Would you implement COVID-19 preventive measures, if this was advised?								
Certainly not	3	0.6%	0	0.0%	0	0.0%	3	0.4%
Probably not	0	0.0%	0	0.0%	0	0.0%	0	0.0%
Perhaps yes-perhaps no	9	1.9%	21	13.0%	3	3.2%	33	4.6%
Probably yes	54	11.5%	51	31.5%	18	19.4%	123	17.0%
Most certainly	402	85.9%	90	55.6%	72	77.4%	564	78.0%

*n* frequency, % percentage

Table 10 shows that the population sample from Egypt scored significantly lower compared with the populations from the other two countries (*P* < .01) for the intention to comply with precautionary measures and implement preventive strategies against the COVID-19 outbreak.

**Table 10:** Differences in mean “intention to implement preventive measures” scores, according to country of origin

Intention to implement preventive measures	Saudi Arabia	Egypt	Jordan	Total	Kruskal-Wallis test	*P* value
	*n* = 468	*n* = 162	*n* = 93	*N* = 723		
Would you implement COVID-19 preventive measures, if this was advised?						
M ± SD	4.82 ± 0.513	4.43 ± 0.712	4.74 ± 0.509	4.72 ± 0.585	Chi-square = 66.779	< .001**
Mean Rank	390.94	278.72	361.42			

*M* mean, *SD* standard deviation

**Significant at the .01 level (two-tailed)

Table 11 reveals a significant positive correlation between the mean “perception of COVID-19 seriousness” scores and the mean “self-efficacy” scores among the entire population sample. Among all participants, a higher mean “perception of COVID-19 seriousness” score was most likely associated with a higher mean “self-efficacy” score.

**Table 11:** Correlation between the perception of COVID-19 seriousness and self-efficacy mean scores

Correlation	Mean seriousness scores	Mean Self-Efficacy scores
Mean seriousness scores		
Pearson’s correlation	1	.366**
Sig. (two-tailed)		< .001
N	723	723
Mean self-efficacy scores		
Pearson’s Correlation	.366**	1
Sig. (two-tailed)	< .001	
*N*	723	723

**Correlation is significant at the .01 level (two-tailed)

Table 12 revealed that the population sample aged 35 to 39 years had significantly higher mean self-efficacy scores than all other age categories; however, the lowest mean self-efficacy scores were reported by individuals 50 years and older (*P* < .05). Females reported a significantly higher mean self-efficacy score than male participants (*P* < .01); however, no difference in mean self-efficacy scores was observed according to marital status. Diploma degree holders scored significantly lower for mean self-efficacy than all other educational levels, whereas bachelor’s degree holders had the highest total self-efficacy mean score (*P* < .01).

**Table 12:** Differences in mean self-efficacy total scores, according to sociodemographic characteristics

Sociodemographics	M ± SD	Mean rank	Test	*P* value
Age				
Younger than 24 years	4.47 ± 0.57	365.51	Kruskal-Wallis Test Chi-square = 13.794	.032*
25–29 years	4.46 ± 0.66	353.28		
30–34 years	4.43 ± 0.63	332.44		
35–39 years	4.59 ± 0.48	398.39		
40–44 years	4.48 ± 0.39	381.05		
45–49 years	4.42 ± 0.54	325.15		
50 years and older	4.39 ± 0.49	313.08		
Gender				
Male	4.40 ± 0.62	338.67	Mann-Whitney *U* test = 49878	< .001**
Female	4.62 ± 0.39	402.57		
Marital status				
Single	4.49 ± 0.53	363.24	Mann-Whitney *U* test = 46984.5	.929
Married	4.47 ± 0.56	361.62		
Education				
School	4.51 ± 0.56	362.5	Kruskal-Wallis test Chi-square = 29.613	< .001**
Diploma	4.25 ± 0.67	291.9		
Bachelor’s degree	4.57 ± 0.48	395.68		
Postgraduate studies	4.55 ± 0.35	372.17		

*M* mean, *SD* standard deviation

*Significant at the .05 level (two-tailed)

**Significant at the .01 level (two-tailed)

Table 13 shows the reported motivating and hindering factors that affect the intention and motivation to implement disease preventive measures. The primary reasons reported by participants driving their willingness to perform certain preventive measures against COVID-19 was a feeling of responsibility toward their own health, followed by preventing transmission to other people and the feeling that coronavirus can be serious (61.8%, 57.7%, and 53.1%, respectively). The primary reason reported for the unwillingness of participants to perform precautionary measures included the feeling that the situation did not apply to them, which was reported by nearly two-fifths of the respondents (38.6%). In addition, more than one-quarter of respondents reflected that the great effort, time waste, and high costs associated with complying with preventive measures drove their unwillingness to comply with preventive measures (28.2%). The third reason was the non-compliance of other people in the surrounding area (27.4%).

**Table 13:** Assessment of the motivating and hindering factors that affect compliance with preventive measures (*N* = 723)

Question	Motivating/hindering factors	*n*	%
Q1-Why would you be willing to perform the preventive measures mentioned?	I am often ill	54	7.5%
	COVID-19 can be serious	384	53.1%
	I feel responsible for my health	447	61.8%
	I think I am at risk for contracting COVID-19	147	20.3%
	I want to prevent the contraction of COVID-19	318	44.0%
	I want to prevent transferring COVID-19 to people around me	417	57.7%
	I trust that the measures help	213	29.5%
	The authorities advise it, so I will do it	192	26.6%
	If I do not take these measures, I may regret it later	144	19.9%
	Other people in my environment will also perform these measures	42	5.8%
	Not applied to me	3	0.4%
Q2-Why would you not be willing to perform the preventive measures mentioned?	I am never ill	27	3.7%
	COVID-19 is not serious	78	10.8%
	I do not find it important	39	5.4%
	I am not worried about my health	30	4.1%
	I do not think I am at risk of contracting COVID-19	81	11.2%
	I do not think that I would transfer the virus to others	72	10.0%
	I doubt whether the measures help	99	13.7%
	Takes too much effort (cost, time, etc.)	204	28.2%
	People in my environment will not perform these measures	198	27.4%
	I feel that too little information is provided about the measures	120	16.6%
	For principle reasons (e.g., Religion or other)	57	7.9%
	Not applied to me	279	38.6%

Up to three answers could be chosen by participants

*n* frequency, % percentage

Table 14 displayed desired information regarding COVID-19 expressed by participants. Most of the study sample reported a desire to receive information about COVID-19 treatment, ways to prevent disease contraction, and the incubation period for the novel coronavirus (58.1%, 49.8%, and 42.7%, respectively). The vast majority of participants reported that they preferred to receive COVID-19 updates from national authorities in their countries, such as the Ministry of Health. Similarly, more than two-thirds of respondents declared that they preferred to receive updates regarding COVID-19 through reports published by national authorities (for example, the Ministry of Health) and via letters from local authorities (68.5% and 52.3%, respectively), whereas the smallest proportion of participants preferred to receive information from local newspapers (6.2%).

**Table 14:** Information needs regarding COVID-19, according to participants (*N* = 723)

Question	Information needs	n	%
Q1-What are the most important topics that you desire information for, at this time?	How COVID-19 is transmitted	270	37.3%
	What is the incubation time for COVID-19	309	42.7%
	What are the symptoms of COVID-19	231	32.0%
	What can you do to prevent contracting COVID-19	360	49.8%
	The chances that you will contract COVID-19	153	21.2%
	The chances that COVID-19 is serious	150	20.7%
	How COVID-19 can be treated	420	58.1%
	I do not need any information	54	7.5%
Q2-Who would you like to provide you with this information about COVID-19 updates?	General practitioner	261	36.1%
	Public Health Service	390	53.9%
	National authorities (for example, the Ministry of Health)	648	89.6%
	I don’t know	39	5.4%
Q3-How would you like to receive this information?	A letter from the general practitioner	93	12.9%
	A letter from the local authority	378	52.3%
	Informational meeting by the Public Health Service	240	33.2%
	National authorities (for example, the Ministry of Health)	495	68.5%
	Information during a talk with the general practitioner	78	10.8%
	Information on the website from the Public Health Service	174	24.1%
	Information on the local authority's website	156	21.6%
	Information in local newspapers	45	6.2%
	Adverts on the national TV/radio	156	21.6%
	Information on an internet search engine (e.g., Google)	126	17.4%

Up to three answers could be chosen by participants

*n* frequency, % percentage

## Discussion

The results of this study revealed that the majority of the study sample were aware that no vaccine is readily available against the novel coronavirus, as COVID-19 is a newly emerging pandemic with insufficient data regarding effective treatment protocols, and no vaccine has yet been developed. Bai et al. [37] reported that in the absence of effective vaccines, medical, and pharmacological treatments, the current social distancing and health-protective behaviors are likely to remain necessary for a long time, especially as many individuals infected with COVID-19 are asymptomatic or have only mild symptoms.

The present study found that the majority of participants perceived the COVID-19 pandemic as being serious or very serious, and the mean scores for all items associated with the perception of coronavirus seriousness, in addition to the total score, were the highest among Saudi participants, followed by Egyptian participants and then Jordanian participants. These differences may be due to the low numbers of confirmed COVID-19 cases and deaths reported in Jordan compared with those in Saudi Arabia and Egypt, which may affect the perception of seriousness among participants. In addition, Saudi Arabia has experienced previous outbreaks of epidemic pathogens, such as SARS and Ebola, which may provide a reference point for evaluating the risk perceptions of the current COVID-19 pandemic.

This result was congruent with the results reported by Kyaw et al. [38], who reported that adults in Myanmar reported moderate to high levels of risk perception regarding COVID-19. However, the scores of risk perception for COVID-19 in the present study were lower than those reported in a study conducted in Hong Kong, by Kwok et al. [39], who reported that individuals in the Hong Kong community had high-risk perception toward COVID-19, high perceived susceptibility, and high perceived severity during the initial stages of the outbreak, due to the disease uncertainty (including transmissibility, route of transmission, and pathogenicity).

The present study demonstrated that nearly half of the study sample reported “perhaps yes-perhaps no” for their perception of the risk of contracting COVID-19 during the coming year without taking any preventive measures, whereas one-fifth responded, “most certainly.” The population sample of Saudi Arabia scored significantly higher for their perception of the risk of contracting COVID-19 in the absence of preventive measures compared with the Egypt and Jordan samples. The majority of participants responded “not small, not large” or “large chance” for the items associated with perceived susceptibility to COVID-19 and the extent of anxiety regarding disease contraction. These differences in the perception of susceptibility may be due to a lack of public awareness regarding the COVID-19 pandemic, the delayed application of precautionary measures in Egypt, and ignoring disease onset, which resulted in the underestimation of the pandemic situation in Egypt, compared with the responses in Saudi Arabia and Jordan. Sawaya et al. [40] demonstrated that Egypt faced a rapid surge in the numbers of COVID-19 cases and deaths because public health measures were announced and implemented late. Lewnard and Lo [41] demonstrated that the evidence suggested the underreporting of COVID-19 cases, for various reasons, such as the lack of surveillance and diagnostic capacities and the lack of medications and treatment protocols for managing COVID-19 cases.

The population sample of Egypt scored lower in all items associated with perceptions of efficacy and self-efficacy, with significantly lower mean self-efficacy total scores than the participants from Jordan and Saudi Arabia. The observed difference among these three Arab countries, with regard to the self-efficacy of protective behaviors, may be attributed to differences in education levels, as more than half of the Egyptian sample had only a moderate educational level (diploma) compared with the higher educational levels reported by participants from the other two countries, which may affect the level of knowledge and risk perceptions regarding COVID-19 and, subsequently, alter the sense of self-efficacy toward COVID-19. Financial difficulties may also play a vital role in participants’ feelings of low self-efficacy to deal with the pandemic, as quarantine causes an economic burden for the majority of the population who work for the private sector. Furthermore, the need to purchase precautionary equipment, including alcohol, detergents, soap, gloves, and masks, for the whole family may present an additional monetary burden for participants from Egypt due to the generally worse financial status and low governmental subsidy.

Leppin and Aro [42] suggested that increased risk perceptions may only predict protective behaviors when people believe that effective protective actions are accessible and readily available (response efficacy) and when they are confident that they have the ability to engage in protective actions and tolerate their conditions (self-efficacy). Despite these countries’ wide and exceptional measures, which have been employed to combat the outbreak, the success or failure of these efforts is generally dependent on public behavior. Therefore, encouraging public adherence to preventive measures is of high significance for preventing the spread of the disease. Adherence is, in all likelihood, influenced by the public’s knowledge and attitudes toward COVID-19. Evidence has confirmed that public knowledge and awareness are necessary to appropriately address pandemics [43]. By assessing public awareness of information regarding COVID-19, deeper insights into existing public perceptions and practices can be gained, allowing those attributes that impact the public willingness to adopt healthful practices and responsive behaviors to be determined [44].

Tang and Wong [45], in their study of adult Chinese individuals in Hong Kong, suggested that compliance with health-related guidelines, such as preventive measures, would likely increase if people believed that they had a high probability of being infected or if they perceived the illness to have a significant negative consequence. Similarly, De Zwart et al. [46], in their international survey, reported that efficacy attitudes toward SARS were more positive in Asia, where people felt more capable of dealing with and controlling SARS. Alternatively, the increased direct and closer experience with SARS in Asia and the unique experience of outliving and overcoming the SARS outbreak may have improved people’s self-efficacy and response efficacy beliefs in Asia. Preventive measures in Asia were also more visible, which may have been more reassuring to the public. In the same vein, Zhong et al. [47] reported that the majority of Chinese residents (98%) had good compliance and practiced appropriate measures for the prevention of COVID-19.

Although the vast majority of the study sample reported a positive intention to perform the advised preventive measures against the novel coronavirus, the population sample from Egypt scored significantly lower for this metric than the populations of the other two countries, which reflects a lower intention to comply with precautionary measures and perform preventive actions against the COVID-19 outbreak. This variation may be due to the dire consequences of COVID-19 in Egypt, which has generally been attributed to the terrible state of the health infrastructure, poverty concerns, detrimental living conditions in cities, excessive population densities, and the prevalence of underlying disease conditions. In addition, confirmed cases in Egypt are not diagnosed until they visit healthcare facilities with serious signs and symptoms of COVID-19. The monetary burdens associated with preventive measures negatively influence work and business, particularly among private-sector workers. In contrast to Egypt, the Saudi Arabian and Jordanian governments strictly applied to WHO protocol, to contain the outbreak, implementing an early public curfew, testing all individuals who showed COVID-19 symptoms, ensuring that all cases who test positive are systematically hospitalized and properly treated, regardless of symptoms, ensuring that COVID-19 contact cases are quarantined, and providing free COVID-19-related medical care for all residents and citizens. Abdelhafiz et al. [48], who conducted a study on the Egyptian population, mentioned that Egypt is one of the largest countries in the Arab region, Africa, and the Middle East (with more than one hundred million inhabitants). This extremely high population could be associated with the increased risk of disease spread and mortality, particularly among older individuals and those with chronic diseases. Global efforts have been made to stop and mitigate the spread of the virus, including political efforts made by governments and changes in personal attitudes and behaviors, which rely heavily on the awareness and knowledge of the general public regarding the disease.

The results of the present study agree with the results of the study reported by Branas et al. [49], who suggested that many deaths are not directly caused by coronavirus but can instead be attributed to the reality that hospitals become overwhelmed and patients are not handled properly. Similarly, Shahnazi et al. [50], in their study in Iran, suggested that the perceived benefits associated with the implementation of preventive actions were factors that could predict compliance with precautionary behaviors against the COVID-19 outbreak. Individuals perform better when the perceived benefits increase. Having positive perceptions of the effects of frequent hand washing and the use of personal protective equipment, such as masks and disposable gloves, can lead to higher perceived benefits, which are robust motivations for complying with preventive measures. Moreover, Jordan et al. [51] found that focusing on “your community” had a positive effect on the intention to engage in numerous preventive behaviors, in contrast to the baseline, which may represent a beneficial suggestion and useful recommendation for leaders and policymakers.

Similarly, Al-Hanawi et al. [13] mentioned that following the WHO declaration of COVID-19 as a worldwide pandemic, countries around the globe, including the Kingdom of Saudi Arabia, began to enact strategic plans that were tailored to mitigate the pandemic spread, reduce daily reported infected cases, increase daily recovered cases, and contain the virus. Following the confirmation of its first case of COVID-19, on 2 March 2020, the Saudi Arabian government enacted effective measures, which were applied throughout the country, under the threat of penal law, to control and prevent the spread of the disease, combined with the registration of new COVID-19 cases and daily follow-up reporting, in accordance with the instructions provided by the WHO for handling this pandemic. Upon confirming the first case in Jordan, however, countrywide measures were scaled-up, to limit and tackle the spread of COVID-19 during the early stages [52].

The Saudi Arabian Ministry of Health (MOH) has enacted an intensive awareness campaign, communicated through its website, television, and social media platforms. The MOH has produced a guide to COVID-19, to offer residents necessary information and precautionary messages, available in more than 10 languages. The MOH has also communicated with the public and the media, through social media platforms. These early intervention efforts to inform the public of prevention and mitigation measures, as well as extensive efforts to counter rumors and misinformation, have been significantly increased as the pandemic continues [53]. The KSA is in a unique position of having dealt effectively with two outbreaks of viral origin, associated with similar viruses [54]. This unique experience has helped the government enact an instant response and implement precautionary measures against COVID-19, to control the disease spread.

The results of the current study revealed a positive significant correlation between the mean scores for the “seriousness perception” of COVID-19 and the mean “self-efficacy” scores, among the entire population sample. Among the study participants, higher seriousness perception mean scores were significantly associated with higher self-efficacy mean scores. Although the COVID-19 epidemic can be perceived as a threatening situation, individuals that confront this hazard in a manner that restores the experience of control may alleviate stress reactions, which, ultimately, can have a positive effect on emotional health and well-being. According to social cognitive theory, Bandura [55] reported that perceived coping efficacy (e.g., the perception that one is capable of managing or coping with a threat) plays a key role in effective adaptation.

Brewer et al. [56] described public health authorities as being dependent on the willingness and capacity of the general public to adhere to disease prevention recommendations, including those associated with personal hygiene, vaccination and/or prophylaxis, quarantine, transportation restrictions, and the closing down of public institutions, such as schools and universities; however, one factor that can affect the willingness and motivation of the public to integrate and adopt precautionary behaviors is risk perception. Qian et al. [57] and Wang et al. [58], in studies performed in China, reported that higher perceived risk regarding coronavirus dangers was positively linked with favorable behavioral responses and substantially higher anxiety levels among the general population.

The results of the current study revealed that the population sample aged 35 to 39 years had significantly higher mean self-efficacy scores than all other age categories, whereas the lowest mean self-efficacy scores were identified among the oldest population (50 years or older), which may be associated with increased worries regarding potential complications associated with COVID-19 and higher mortality rates, especially among those with chronic diseases.

Similarly, the current study revealed that females scored significantly higher for mean self-efficacy scores compared with males, and those with bachelor’s degrees had significantly higher self-efficacy mean scores than all other educational levels, which may be due to their abilities to obtain more information and gain more insight into COVID-19. Similarly, Lau et al. [59], who studied the hemagglutinin type 1 and neuraminidase type 1 swine flu (H1N1) pandemic in Hong Kong, found that females had better overall performance than men for disease prevention. Furthermore, Guo et al. [60] reported that older individuals and patients with comorbidities are more likely to become infected with COVID-19 and are more prone to developing serious complications, such as acute respiratory distress syndrome and cytokine storm, placing them under greater psychological stress.

Similarly, El-Zoghby et al. [61], in their study in Egypt, reported that higher educational levels were associated with higher awareness, which can increase participation in preventive measures and precautionary practices in cases of suspected infection, limiting their feelings of stress. Abdelhafiz et al. [48] demonstrated that individuals with university-level or higher educations had drastically greater awareness mean scores regarding COVID-19 compared with participants with lower levels of education.

Studies in Hong Kong revealed that those with higher levels of education were more likely to undertake precautionary behaviors to defend against SARS [62] and avian influenza [63], including frequent handwashing, respiratory hygiene, mask-wearing, the proper use of utensils, and hand washing after touching contaminated surfaces. More educated individuals in Australia also reported a higher intention to wear face masks during pandemic influenza events [64].

Participants’ willingness to perform preventive measures against COVID-19, in the current study, was primarily driven by a feeling of responsibility toward their own health, followed by the desire to prevent coronavirus transmission to other people, and the feeling that coronavirus can be serious. The perception of personal infection risk and the perceived seriousness of the health-related consequences have both been linked to engagement with disease-preventive behaviors. Because COVID-19 is spread relatively rapidly by direct human-to-human contact, fighting this disease has been more challenging and has required governments to inform the public of the risks and necessary precautions for protecting themselves and others. However, the feeling of personal responsibility was evidenced in the Arab culture.

This result was congruent with the results of Bish and Michie [65] who reported that higher perceived personal risk predicts an individual's engagement with disease-preventive behaviors, such as hand washing and social distancing, as reflected by studies of prior pandemics. Similarly, Abdelhafiz et al. [48] reported that most of their study participants believed that COVID-19 represents a life-threatening hazard and have been worried about the possible risk of COVID-19 infections among members of their families.

When asked why they may not be willing to undertake preventive measures, in the current study, almost two-fifths of participant reported that the situation does not apply to them, and slightly more than one-quarter answered that the measures require too much effort (cost, time, etc.) and that the people in their environment would also not perform these measures. These responses reflect the public desire to obtain accurate and adequate information regarding the nature of the novel coronavirus disease pandemic and the necessity of orienting and educating the public regarding the importance of utilizing and abiding by the suggested preventive measures to control COVID-19 transmission. Similarly, van der Weerd et al. [66], in a study performed in the Netherlands, reported that receiving information from a variety of sources during the course of the new influenza H1N1 pandemic, such as public health professionals, the government, and the media, could expand people’s awareness of risks and, consequently, increase their adoption of the recommended preventive measures.

The current findings suggested that most of the study sample reported their desire to receive information regarding COVID-19 treatment, methods of preventing disease contraction, the incubation period of the novel coronavirus, and how COVID-19 is transmitted. Because COVID-19 is a newly emerging disease, with no understood natural history and no definite treatment or vaccine, the community required knowledge regarding the etiological agents, epidemiological parameters, such as the virus incubation period, transmission modes, signs and symptoms, and preventive measures. Alzoubi et al. [67] and Huang et al. [68], who conducted studies in Jordan and Wuhan, respectively, reported that no specific antiviral drugs or vaccines have yet been identified to treat COVID-19. Al-Hanawi et al. [13] reported that approximately half of their study respondents were unaware that SARS-CoV-2 could transmit person-to-person in close proximity. Therefore, the general population of that study had a low level of awareness of when protective masks were necessary and who must use them to prevent disease transmission.

The results of the current study demonstrated that the vast majority of participants reported that they prefer to receive COVID-19 updates from national authorities of their respective countries, such as the Ministry of Health. More than two-thirds of the respondents declared that they prefer to receive COVID-19 updates through the reports published by national authorities and letters sent by local authorities, indicating that people actively choose which resources they trust for information. Providing information through various sources can play an important role in managing threats by influencing the public’s judgments of risks and related benefits, which may indirectly impact the adoption of recommended measures. However, trust is a core component for the public to listen to, understand, and react to public health messages.

Several studies have mentioned that during an epidemic, understanding how important information regarding health hazards is disseminated and how the public accesses, processes, understands, and uses this information is crucial [69, 70]. The threats and uncertainties associated with emerging infectious diseases may additionally arouse public emotions and alter behaviors in constructive (e.g., engaging in personal hand hygiene and averting mass gatherings) or disruptive manners (e.g., inflated public fear, useless anxiety, and socioeconomic unease) [71]. Xiao et al. [72] reported that during quarantine, the separation and restriction of movement among the population fall within the mandate of the government and public health authorities. Therefore, having confidence in the judgments of government and public health authorities can have remarkable effects on the intellectual health and well-being of individuals under quarantine. Individuals who self-isolated during the COVID-19 virus epidemic in central China who had more family support and higher social trust reported lower levels of anxiety and stress. Sawaya et al. [40] also mentioned that governments and the WHO have resorted to TV, social media outlets, and cellular operators to disseminate information to increase public awareness about COVID-19 and promote safe physical distancing while combatting the disease outbreak in the Middle East.

## Conclusion

The perception of COVID-19 seriousness was significantly higher among participants from Saudi Arabia and the perception of susceptibility to COVID-19 and the extent of anxiety among Saudi Arabians were also high compared with participants from Egypt and Jordan. In contrast, participants from Egypt had a lower perception of efficacy and self-efficacy for dealing with COVID-19 and performing preventive measures. Moreover, Egyptian participants reported a lower intention to comply with the recommended precautionary measures against COVID-19.

A significant positive correlation was detected between participants’ perception of COVID-19 seriousness and their self-efficacy to handle COVID-19, across the whole sample. The main driving forces for participants to implement the recommended preventive measures included their feelings of responsibility toward their own health, followed by preventing coronavirus transmission to other people, and the feeling that coronavirus can be serious. The general public requires information regarding COVID-19 treatment, disease prevention methods, and the incubation period of the novel coronavirus from reliable resources, such as the ministry of health and local authorities.

### Recommendations

Communications via various channels designed to promote the adoption of preventive behaviors among the public should focus on raising the public’s perceptions of disease seriousness and risks and promote the effectiveness of adopting behavioral measures to reduce the perceived risks, to improve the self-efficacy of COVID-19 outbreak control. Health education programs that aim to improve COVID-19 knowledge and perception are recommended to increase public awareness and compliance with advised preventive measures. These findings highlight the need to continue encouraging and emphasizing the maintenance of social distancing, hand hygiene, and wearing masks to prevent the spread of the virus. The development of structured psychological interventions is necessary to support the population and to combat the psychological impacts and anxieties associated with the COVID-19 pandemic.

### Limitations

Randomization and cross-sectional design were the major limitations and threats to the study generalization; therefore, similar future studies should be performed, using longitudinal research designs, with the inclusion of random samples from more countries, and using a larger sample size. Non-random sampling is among the factors which jeopardize the internal validity of the study and might brought about a difference in the variables of COVID-19 risk perception (perception of disease susceptibility, perception of COVID-19 seriousness, and perception of efficacy and self-efficacy to cope with COVID-19) among the samples of the three countries, e.g., the educational level for the sample of Egypt was markedly lower than that for others.

## Data Availability

The datasets of the current study are available from the corresponding author on reasonable request.

## Abbreviations

COVID-19: Coronavirus disease 2019
SARS-CoV-2: Acute respiratory syndrome coronavirus 2
MERS: Middle East respiratory syndrome
WHO: World Health Organization
SCDC: Saudi Center for Disease Prevention and Control
SPSS: Statistical Package for Social Sciences
MOH: Ministry of Health
H1N1: Hemagglutinin type 1 and neuraminidase type 1 swine flu
